# Evaluating the Sociocultural Barriers Impacting Cervical and Breast Cancer Screening Among Nepali and Bhutanese Women in the Dallas-Fort Worth Metroplex

**DOI:** 10.1007/s10900-026-01611-9

**Published:** 2026-07-25

**Authors:** Madeline Neff, Allison Powell, Kristina Kafle, Catalina Beltran, Sara Al-Sammarie, Melody Nguyen, Rafael do Valle, Kristen Miguel, Amany K. Hassan

**Affiliations:** Texas College of Osteopathic Medicine, University of North Texas Health Science Center, Fort Worth, TX USA

**Keywords:** Cervical cancer screening, Breast cancer screening, Nepali immigrants, Reproductive health, Health disparities, Health Belief Model, Women's health

## Abstract

Despite widely accessible screening, cervical and breast cancer remain prevalent diseases, especially amongst Nepali women. The Dallas-Fort Worth Metroplex in Texas is home to one of the largest Nepali immigrant communities in the United States. This study seeks to understand barriers preventing this population from accessing preventative reproductive healthcare. A mixed-methods survey assessing health beliefs and reproductive cancer screening utilization was completed in English by 58 women and in Nepali by 4 women. Despite most participants reporting knowledge of the importance of reproductive cancer screening (94% breast cancer, 87% cervical cancer, 74% HPV), the rates of receiving obstetric and gynecologic care in the past year (57%), receiving a cervical exam (42%), breast exam (29%), and history of HPV vaccination (47%) were low in comparison. One-way ANOVA showed a significant difference in reproductive health knowledge and having ever seen an OBGYN (p = .018). Education did not correlate with cancer screening behavior as measured by the Health Belief Model (Susceptibility: p = 0.091, Severity: p = 0.141, Barriers: p = 0.38, Benefits: p = 0.22). Free-response results yielded comments about modesty, cultural competency, and praise for the American healthcare system as it compares to foreign medical systems. There is a discrepancy between the DFW Nepali immigrant community’s reproductive knowledge and their reproductive healthcare utilization. Healthcare providers in DFW should consider exam modification and community outreach to encourage Nepali women to receive cancer screenings and ultimately reduce morbidity and mortality of reproductive cancers in this population.

**Supplementary Information:**

The online version contains supplementary material available at https://doi.org/10.1007/s10900-026-01611-9.

## Background

Despite medical advances in women’s health throughout the twenty-first century, cervical cancer remains the fourth most common cancer globally [1]. In Nepal, breast and cervical cancers have historically represented the two most prevalent malignancies among women, with cervical cancer carrying a particularly significant sociocultural burden [1]. Low screening rates have been attributed in part to stigma surrounding cervical cancer, as it is associated with sexually transmitted infections [2]. In fact, only 8% of Nepali women aged 30–49 were screened for cervical cancer in 2019 [2] despite all women in this age range being recommended to complete screening. Compounding this stigma are cultural practices such as the Nepali practice of Chhaupadi, in which menstruating women are subjected to restrictions including exclusion from the home, limited dietary intake, and social isolation [3]. Although banned in 2005 and criminalized in 2017, this practice persists in some rural regions of Nepal [4]. These conditions have been associated with poor hygiene, undernutrition, and increased susceptibility to infection, including human papillomavirus (HPV) [3]. Consequently, cervical cancer has been the most common cancer among women in Nepal with a 30% increase from 1990 to 2017 [5, 6]. 

More recent epidemiologic data, however, suggest a shift in disease burden. Surpassing cervical cancer in recent estimates, breast cancer now represents the leading malignancy among women in Nepal, accounting for approximately 19.1% of all cancers [5]. Breast cancer is the third leading cause of cancer-related mortality globally, contributing to 12.3% of cancer deaths among women [5]. Notably, a higher incidence of breast cancer was observed in urban populations compared to suburban and rural regions, reflecting the influence of urbanization and lifestyle changes on breast cancer incidence and detection [5]. Although incidence rates remain lower than in neighboring countries, such as India and China, the burden of breast cancer in Nepal continues to rise, with a lifetime risk estimated at approximately 1 in 76 women [5].

The United States receives millions of immigrants annually, more than 1000 of them being Nepali. Similar to other immigrant populations, Nepali immigrants arrive in the US with social, cultural, and economic stressors preventing them from accessing healthcare [7, 8]. Although the US has lower rates of cervical cancer compared to Nepal, the risk of preventable cancer remains high in immigrant populations. South Asian immigrants are more than three times as likely to lack cervical cancer screening (22.8%) compared to US-born women (6.8%)—a concerning disparity given that screening rates are the main predictor of cervical cancer mortality [9, 10].

Several barriers to cancer screening have been previously identified among immigrant and refugee women, such as one’s income, education level, age, and marital status [11]. Additionally, a 2021 study titled Factors related to cervical cancer screening among Asian women asserts that both cultural subgroup and individual beliefs are critical factors to consider when addressing cervical cancer screening hesitancy [12]. Immigrants to the US face a unique set of challenges accessing and utilizing healthcare in addition to the obstacles impacting the American population, including “provider gender mismatch, providers who are insensitive to cultural or religious beliefs, and lack of language interpreters” [11].

In addition to cultural barriers that Nepali women may face, there are numerous barriers that many patients experience in the US due to the current state of the healthcare system. These include insurance coverage, prior authorization requirements, cost, technical literacy, fear of racism/bias from providers, and lack of appointment availability, among other barriers commonly cited. Each of these barriers holds its own burden but cost alone prevented an estimated 42% of insured Americans making less than $25 k annually and 12% making over $100 k annually from seeking care in 2023 [13]. This effect compounds when considering prescription costs, follow-up appointment copays, and adjuvant therapies like physical therapy that may be included in treatment.

The aggregation of Asian populations in health research has been increasingly recognized as a limitation with clinical consequences [14, 15]. South Asia encompasses eight countries: Afghanistan, Bangladesh, Bhutan, India, the Maldives, Nepal, Pakistan, and Sri Lanka. Yet, these populations are frequently treated as interchangeable in both research and clinical settings [15]. The present study focuses on two distinct but interconnected communities: Nepali women and Nepali-speaking Bhutanese women in the United States. Though they share linguistic and cultural ties, their pathways to the US differ significantly [16, 17]. Nepali women have immigrated largely through economic and educational channels, while the majority of Nepali-speaking Bhutanese women are Lhotshampa — an ethnic Nepali population expelled from Bhutan in the early 1990s — who arrived in the US as refugees following prolonged displacement in UNHCR camps in southeastern Nepal [16–20]. Despite these distinct histories, in the US context, these communities are frequently co-located, share community organizations and cultural infrastructure, and are routinely grouped together by healthcare systems and researchers alike.

This study includes both populations, not to generalize them, but because their lived experience in the US is interwoven. Understanding their reproductive health needs requires engaging with this reality rather than imposing an artificial separation. By examining these communities together while remaining attentive to their distinct backgrounds, this study aims to surface the nuanced, within-group variation that aggregated research consistently misses. Because the Dallas-Fort Worth metropolitan complex is home to the largest Nepali and Bhutanese immigrant population in the country [21], it is beneficial to survey this community in order to better understand and meet their needs. Therefore, this study aims to address perceptions of cervical and breast cancer screenings held by Nepali women in the Dallas-Fort Worth metroplex.

## Study Objectives

Describe the perceived knowledge of breast and cervical cancer among Nepali women.

Describe the perceived risk of breast and cervical cancer, and the perceived value of screening among Nepali women based on their health beliefs.

Identify barriers to cervical and breast cancer screening among Nepali women.

Analyze the association between health literacy and frequency of OBGYN visits.

## Methods

### Study Design, Sample, and Theoretical Framework

This is a cross-sectional study using an online survey to measure the perceived value and barriers to cervical and breast cancer screening. Participants included Nepali women residing in the DFW metroplex. Access to the Qualtrics survey was shared at The Nepali International Church, the University of Texas at Arlington, and the University of Texas at Dallas. These locations were chosen because they have high concentrations of Nepali members. All respondents signed informed consent in their preferred language, English or Nepali, approved by the [Institution name redacted for review]. Participants were recruited through advertisements using a flyer created for this study, both at the church and via email sent through the Texas Nepali Student Associations of the two universities. A novel, 20-min survey (Appendix Item 1) was created to assess the demographic, healthcare history, and cultural beliefs of Nepali women in DFW. The Health Belief Model (HBM) was the theoretical framework used in the survey to measure the perceived current health of respondents, the perceived risk of breast and cervical cancer, and the perceived value of screening based on their health beliefs [21]. The survey consisted of six primary cognitive constructs: perceived susceptibility, perceived severity, perceived benefits, perceived barriers, self-efficacy, and cues to action. The survey domains included questions in each of these constructs concerning breast and cervical cancers. The survey also collected demographic and other baseline characteristics such as age, marital status, level of education, access to healthcare, and number of years in the US. Questions were a variety of free-response and five-point Likert scale-style questions. The survey was independently assessed for face validity by four members of our research team. Questions were revised based on their feedback.

### Statistical Analysis

Descriptive statistics were used to summarize the characteristics of the study participants. Means and standard deviations were used for continuous variables, including Likert scale items, while frequencies and percentages were used for categorical items.

For the analysis, we used two sample t-tests to compare differences in the mean Likert scale of the HBM domain scores between groups of participants based on education status. To achieve this, participants were grouped into two main groups: Lower education level (less than high school, high school) vs higher education group (some college, two-year degree, four-year degree, professional degree, doctorate).

Also, we conducted one-way ANOVAs to compare the knowledge of participants related to cervical and breast cancer and HPV among three groups: never been to an OBGYN, been to an OBGYN in the past 3–10 years, or been to an OBGYN in the past year.

All data analyses were conducted using SPSS V 30.0.0.0 (172) (IBM Corp, 2024) [22]. Significance was set at an a priori alpha level of 0.05.

For qualitative, free-response items, we noted thematic trends in responses as they pertain to an individual’s likelihood to seek reproductive care. Two investigators examined the comments independently (MN and AP), summarized themes, and then met to reach consensus over the results.

## Results

### Demographics

While 58 individuals took the survey, not all participants answered every question. The percentages reported in the text are item-level percentages (denominators reflect the number of respondents who answered each specific item). The four survey responses completed in Nepali were excluded from analysis due to incomplete responses. 32.8% (n = 19) were aged 18–25 years and 25.9% (n = 15) were aged 26–30 years. Nearly half (n = 27, 54%) reported being married. Educational attainment was high, with 36.0% (n = 18) reporting completion of a four-year degree, 14% (n = 7) holding a professional degree, and 18% (n = 9) reporting some college. The majority of respondents were US citizens (n = 35, 74%), with 56% (n = 27) reporting dual citizenship with Nepal. Most respondents (n = 35, 71%) had private health insurance, while 12% (n = 6) reported coverage through Medicare or Medicaid.

### Health Status and Utilization

Most respondents had access to healthcare (n = 44, 75.9%). The majority of respondents (n = 37, 77%) had seen a healthcare provider of any type within the past year; however, fewer had seen an OBGYN specifically (n = 27, 57%). Only 42% (n = 20) reported a cervical or vaginal examination, and 29% (n = 14) reported a breast examination or mammogram within the past year. History of HPV vaccination was reported by 47% (n = 22) of respondents.

### Health Knowledge and Perceptions

Nearly all respondents reported awareness of breast cancer (n = 44, 94%) and cervical cancer (n = 41, 87%), while fewer reported knowledge of human papillomavirus (HPV) (n = 35, 74%). The majority of respondents reported having access to healthcare (n = 44, 92%) and feeling that they had control over their health (n = 45, 94%).

Women who had a visit to the OBGYN in the year had significantly higher mean cervical health and HPV knowledge scores, as measured by the five-point Likert scale questions, compared to women who never visited an OBGYN or who had a visit more than a year ago. Overall, the knowledge scores were higher for the breast cancer knowledge domain compared to the cervical cancer or HPV knowledge domains. See Table 1.

**Table 1:** Differences in mean knowledge scores of women’s health screening domains by time since most recent OBGYN visit (n = 46)

Knowledge domain scores, mean (SD)*	Seen an OBGYN			p-value
	Never (SD)	More than a year (SD)	Within the past year (SD)	
Cervical Health Knowledge	3.71 (1.14)	4.33 (1.21)	4.62 (0.57)	0.012^+^
HPV knowledge	3.00 (1.30)	4.00 (1.10)	4.15 (1.12)	0.017^+^
Breast cancer knowledge	4.21 (1.05)	4.50 (1.22)	4.69 (0.68)	0.270
Total knowledge score***	3.64 (0.97)	4.28 (1.14)	4.49 (0.71)	0.018^+^

^*^Mean score of knowledge on a 5-point Likert scale question. Higher scores indicate higher knowledge of female reproductive cancers. **One-way ANOVA. ***Total knowledge was calculated by summing the three health knowledge scores above. ^+^p < 0.05

### Health Belief Model

While the Health Belief Model has six constructs (Perceived severity, perceived benefits, perceived susceptibility, perceived barriers, cue to action, and self-efficacy), this study focuses on the four constructs that regard social barriers. Respondents’ mean scores were low for the four constructs evaluated. The lowest mean was in the “severity” construct (average 1.94 in lower education, 1.55 in higher education), while the highest was in “benefits” (3.36, 3.66). There were no significant differences in mean scores by level of education See Table 2.

### Social Barriers

Most respondents reported trusting healthcare providers in the context of a well-woman exam (n = 42, 87%). A small minority reported participation in Chhaupadi (n = 3, 5.1%). Beliefs about self-management of cancer varied: 25% believed they could cure their own cancer through prayer or another spiritual method alone (n = 11), and 43% believed they could cure their own cancer through lifestyle changes alone (diet, exercise, etc.; n = 19). Embarrassment about a cancer diagnosis was uncommon yet was nominally increased in reproductive cancers compared to skin cancer: 7% for skin cancer (n = 3), 13% for breast cancer (n = 6), and 16% for cervical cancer (n = 7).

Preferences for provider gender and exam acceptability were strong: 88% preferred female OBGYNs/women’s health providers (n = 38), and 83% reported they would be more likely to consent to a breast or cervical exam from a female doctor (n = 35). Nonetheless, a minority indicated modesty-related refusals even when their preferred provider was available: 15% would decline breast exams to stay modest (n = 6) and 16% would decline cervical/vaginal exams to stay modest (n = 7). Perceptions of exam pain varied by exam type: 51% rated Papanicolaou smears (cervical exams) as painful (n = 21), 34% rated vaginal exams without Pap as painful (n = 14), and 22% rated breast exams as painful (n = 9) Table 2.

**Table 2:** Differences in the Health Belief Model Constructs by level of education (n = 41 – 46)

		Education*		
		Lower education,	Higher education,	
HBM construct	n	mean (SD)	mean (SD)	p-value
Perceived Cancer Susceptibility	45	2.97 (1.23)	2.40 (0.84)	0.091
Perceived Cancer Severity	45	1.94 (0.87)	1.55 (0.71)	0.141
Perceived Screening Barriers	46	2.92 (1.17)	2.63 (0.85)	0.38
Perceived Screening Benefits	41	3.36 (0.75)	3.66 (0.55)	0.22

^*^Education: Lower education group: less than high school, high school. Higher education group: some college, 2-year degree, 4-year degree, professional degree, doctorate

## Free Response Questions

Twelve women completed the optional free-response questions included in the survey in English, and two women completed the survey in Nepali. Thematic analysis was conducted by two independent reviewers (MN and AP).

### Cultural and Spiritual Considerations in Women’s Healthcare

When surveyed on sensitivity to cultural and spiritual beliefs during women’s health visits, the majority of respondents indicated that their beliefs did not affect their care. However, a few participants described concerns related to modesty and religious identity, particularly among women practicing Hinduism. One participant noted that while her own Christian faith had not affected her care, she fears her Hindu friends “feel inadequate or different to receive care.”

### Perceived Differences in Women’s Health Care between the United States and Abroad

Participants frequently described differences between women’s health care in the US and care received internationally, particularly in Nepal. One respondent reported that “outside of the US it is a lot harder to receive women’s health care, and shameful.” Another reported that Nepali physicians were “short, harsh, and dismissive of pain.” In contrast, the US-based care was described as more patient-centered, with respondents highlighting greater provider friendliness, transparency, cleanliness, and respect for patient autonomy. One respondent stated that the US is “more careful compared to out of states,” while another stated that women can “talk freely here in the US rather than Nepal.” Some participants also noted that while care was less expensive abroad, they perceived higher quality communication and patient engagement in the US.

### Experiences during Women’s Health Examinations

Responses regarding women’s health examinations varied widely. Some participants reported generally positive experiences with no major concerns. Others described discomfort during pelvic examinations and Pap smears, particularly when having to “repeat the exam due to insufficient samples and it is uncomfortable.” A small number of respondents reported distressing experiences, including a lack of provider sensitivity to pain and refusal to accommodate requests for female providers. One respondent described her experience, stating that she “cried for a woman doctor during a pelvic exam and they refused to get me one since there weren’t many female doctors on staff.” Another respondent shared that she is a survivor of sexual assault, and as such, finds pelvic exams “extremely triggering.” These respondents emphasized the importance of provider awareness of pain, discomfort, and patient vulnerability during intimate examinations.

## Discussion

This study examined sociocultural barriers affecting Nepali and Bhutanese immigrant women in receiving women’s health screenings, specifically for breast and cervical cancer. Prior research conducted in Nepal demonstrates similarly low screening uptake despite awareness of cervical cancer. A systematic review by Shrestha et al. found that cervical cancer screening utilization remains limited, with barriers including lack of knowledge regarding screening procedures, cultural stigma, and healthcare access challenges. Mixed methods work by Dangal et al. further identified infrastructure limitations and policy implementation gaps within Nepal’s tertiary hospital systems, reinforcing that awareness alone does not translate into preventive engagement [5, 6].

Screening disparities among immigrant women have also been well documented within the US. Endeshaw et al. reported lower cervical cancer screening rates among women born outside the US, with screening uptake influenced by duration of residence and healthcare access. Similarly, Elmore et al. identified language barriers, cultural beliefs, and healthcare navigation challenges as significant predictors of screening participation among refugee women attending international clinics [9, 11].

Overall, respondents demonstrated high general awareness of breast and cervical cancer. In our study, most participants reported knowing about breast cancer (94%) and cervical cancer (87%), suggesting that public health messaging and screening campaigns targeting these conditions may be reaching this population effectively. However, knowledge of HPV was comparatively lower (74%), despite HPV’s direct causal relationship with cervical cancer. This discrepancy suggests that while women may recognize disease outcomes, they may be less familiar with underlying etiologies and prevention strategies, including vaccination. Consistent with this, fewer than half of respondents reported HPV vaccination, indicating an actionable gap between awareness and preventive behavior.

Healthcare utilization patterns further contextualized these knowledge findings. While most respondents had seen a healthcare provider within the past year, fewer had seen an OBGYN specifically, and even fewer reported receiving cervical or breast examinations. This drop-off between general healthcare access and reproductive-specific care highlights a structural gap in preventive service delivery. It suggests that simply accessing healthcare does not guarantee receipt of gender-specific screening and counseling, reinforcing the importance of specialty care engagement. This is further supported by our finding that knowledge outcomes differed significantly by OBGYN visit history. Women who had seen an OBGYN, particularly within the past year, demonstrated higher total reproductive health knowledge, cervical cancer knowledge, and HPV knowledge compared with those who had never seen an OBGYN. These findings underscore the educational role of OBGYN encounters beyond clinical screening alone. Preventive visits appear to function as key opportunities for counseling, risk communication, and vaccine education.

Interestingly, while breast health knowledge followed a similar upward trend among women with OBGYN exposure, differences were not statistically significant. This may reflect broader dissemination of breast cancer awareness through media campaigns, primary care counseling, and community outreach, making knowledge less dependent on specialty care exposure.

According to ACOG guidelines, screening mammography is generally initiated at age 40 for average-risk women [23]. This helps contextualize the gap between those with breast cancer knowledge and those who reported receiving a breast examination or mammogram in the past year. However, younger women may still receive clinical breast examinations during routine visits. This discrepancy highlights a potential educational misunderstanding that OBGYN providers can address through anticipatory guidance and age-appropriate screening counseling.

The Health Belief Model provides a useful framework for understanding screening behaviors, suggesting that perceived susceptibility, severity, benefits, and barriers influence preventive health engagement [21]. However, the lack of significant differences in HBM constructs by education level in this study suggests that culturally embedded beliefs and lived healthcare experiences may exert stronger influence than formal education alone. Studies examining Asian women’s screening behaviors have identified modesty, stigma, and fatalistic beliefs as barriers to cervical cancer screening participation. In Nepal specifically, entrenched cultural practices such as Chhaupadi have been shown to influence reproductive health perceptions, reinforcing stigma surrounding gynecologic care and bodily autonomy [3, 12]. Qualitative responses provided additional depth to these quantitative findings. Many participants reported minimal cultural or spiritual considerations affecting their care; however, some described modesty concerns and religious influences, particularly among Hindu women. These insights emphasize the need for culturally sensitive clinical environments that acknowledge modesty, gender preferences regarding providers, and spiritual context.

Participants also described perceived differences in women’s healthcare between the United States and care received abroad, especially in Nepal. US-based care was characterized as more patient-centered, respectful, and hygienic, whereas international care was sometimes described as dismissive of pain or emotionally harsh. These perceptions highlight how communication style and provider empathy shape patient trust and engagement, independent of clinical outcomes.

Experiences during pelvic examinations revealed another critical dimension of care quality. While some participants reported neutral or positive encounters, others described significant distress, particularly when examinations were painful, repeated, or conducted without trauma-informed communication. Survivors of sexual assault reported pelvic exams as especially distressful, reinforcing the necessity of trauma-informed practices, patient consent, and sensitivity to psychological vulnerability during intimate procedures.

This study had several limitations, including small sample size, response bias, and limited generalizability. The number of participants was modest relative to the broader Nepali and Bhutanese immigrant population in the DFW metroplex. As with any self-reported assessment, respondents may have provided inaccurate responses due to recall error or social desirability bias. Additionally, the sample was composed of women recruited through the Nepali International Church and university settings, populations that skew younger and more highly educated, limiting applicability to older or less formally educated immigrant women.

Of note, while prior studies have examined only cervical cancer screening barriers in Nepal and among immigrant populations broadly, the present study contributes a mixed-methods perspective focused specifically on Nepali and Bhutanese immigrant women in the United States assessing both cervical and breast cancer screening. By integrating quantitative knowledge assessments with qualitative reflections on clinical experiences, modesty norms, and trauma-informed care, this work expands beyond screening utilization metrics alone. Our study highlights the relational and experiential dimensions of reproductive healthcare engagement, which emphasizes how provider communication, cultural humility, and patient dignity influence screening adherence. These findings provide actionable insights for clinicians and public health practitioners seeking to provide culturally responsive interventions within Nepali immigrant communities.

## Conclusion

This study highlights that improving cervical and breast cancer screening among Nepali and Bhutanese immigrant women requires more than access to care alone; rather, it depends on addressing the complex interplay between knowledge, cultural beliefs, and healthcare experiences. Despite baseline awareness of female reproductive cancers, a clear disconnect persists between knowledge and screening adherence, underscoring the need for culturally responsive education, trust-building, and trauma-informed, patient-centered care. Encounters with OBGYN providers represent critical opportunities to enhance reproductive health literacy, yet gaps in preventive counseling and sensitivity to sociocultural factors, such as modesty norms, stigma, and prior negative experiences, continue to limit engagement. These findings suggest that effective interventions must integrate cultural humility, community engagement, and system-level changes, including linguistically appropriate resources and strengthened care coordination. Future studies could have focus groups with Nepali women in Nepali and English to further understand cultural nuances influencing reproductive healthcare decision-making in this community. Ultimately, advancing equitable women’s health outcomes will require expanding our cancer screening framework to broader immigrant populations and aligning clinical care with culturally informed public health strategies.

## Supplementary Information

Below is the link to the electronic supplementary material.

Supplementary file1 (DOCX 39 KB)
