# Supplementary material for: Evaluating the Sociocultural Barriers Impacting Cervical and Breast Cancer Screening Among Nepali and Bhutanese Women in the Dallas-Fort Worth Metroplex
Source: J Community Health. 2026 Jul 25;51(6):957–64. doi: 10.1007/s10900-026-01611-9 (PMC13748299; doi:10.1007/s10900-026-01611-9)
Supplement: Supplementary file 1 [file 10900_2026_1611_MOESM1_ESM.docx]

**Appendix A. Survey Instrument**

**Understanding how Nepali culture impacts rates of cervical and breast screening**

**Start of Block: Consent**

Research Study Participation Consent Form Study	Title: Evaluation of Socio-Cultural Barriers Affecting Cervical and Breast Cancer Screening among Nepali and Bhutanese Women in the DFW Area	Principal Investigator: Amany Hassan, PhD Co-Investigators: Madeline Neff, Allison Burkes, Kristina Kafle, Catalina Beltran, Rafael do Valle, Melody Nguyen, Kristen Miguel, Sara Al-Sammarraie Institution: University of North Texas Health Science Center You are being invited to participate in a survey to assess Nepali and Bhutanese women’s perceptions of reproductive health screenings. This research study conducted by the University of North Texas Health Science Center is completely voluntary. If you consent, you will complete this survey online, which will take about 20 minutes. Although there is a chance your survey answers could be shared or stolen, these risks are minimal. Your information will not be tied to any identifying information like your name or email, and all data is kept behind password walls. For a complete description of risks, refer to the detailed consent that follows. While there is no direct benefit for participating in this study, participation will help our investigators work towards better healthcare administration to Nepali and Bhutanese women. You do not have to participate in this study.	**INTRODUCTION/STUDY PURPOSE:** We are conducting a research project to assess Nepali and Bhutanese women’s perceptions of reproductive health screenings.	You are invited to participate in this research study because you are a woman of Nepali or Bhutanese descent. Your participation in this study is entirely voluntary. This consent document contains information to help you decide whether or not you wish to participate. Please read this consent document at your own pace. It is important that you read what is written below and ask questions about anything you do not understand (contact information for the study investigator(s) is included below).	This study will consist of a brief survey assessing your background, your experience around reproductive health screenings (cervical and breast exams), and your perceptions of breast and cervical cancer. If you decide to participate in this study, the survey contains 76 items and will take you approximately 20 minutes to complete	If you are a student or employee at the University of North Texas Health Science Center at Fort Worth, your participation (or non-participation) will in no way affect your academic standing or employment status.	**RISK(S)/BENEFIT(S):** Although minimal, there is a certain amount of risk that is involved with participating in this research study. This study involves informational risks (such as breach of confidentiality). Survey information has the possibility of being stolen or lost, and the survey may include uncomfortable topics for some. Every precaution will be taken by the research team to minimize these risks and ensure your safety. Please remember that if you feel uncomfortable about any of these risks, discuss your concerns with the research team. You do not have to enroll in this study. You may receive no direct benefit from participating in this study. The investigators hope that the information that is gained in this study will benefit healthcare administration to Nepali and Bhutanese women.	**AGREEMENT TO PARTICIPATE:** Participation in the study is completely voluntary. Refusal to participate will involve no penalty or loss of benefits to which you are otherwise entitled. If you decide to participate, you can click the “next” arrow at the bottom of this document to complete the survey.	**CONFIDENTIALITY:** You will not be asked for your name or any other identifying information for the purposes of this study.	**LEAVING THE STUDY:** Since no identifiers will be collected as part of the study procedures, there will be no way to withdraw from the study once you complete it.	**QUESTIONS/CONCERNS:** If you have any questions regarding this research project, please feel free to contact the principal investigator of our study. Principal Investigator: Amany Hassan, PhD Email: Amany.Hassan@unthsc.edu	If you have any questions about your rights as a research participant, please contact the North Texas Regional Institutional Review Board at (817) 735-0409. For more information about participating, or volunteering in research, please visit the NTR IRB website (https://www.unthsc.edu/north-texas-regional-irb/) and click on “For Research Participants” tab. You may also use the following QR code:	Thank you for participating in the study. By clicking the “next” arrow, I am agreeing to participate in this research survey.

**End of Block: Consent**

**Start of Block: Demographics**

Q1 I haven’t filled out this survey before.

o Yes (1)

Q2 Age

o 18-25 (4)

o 26-30 (5)

o 31-40 (6)

o 41-50 (7)

o Older than 60 (8)

Q3 Marital Status

o Married (1)

o Widowed (2)

o Divorced (3)

o Separated (4)

o Never married (5)

Q4 Employment Status

o Employed Full Time (1)

o Employed Part Time (2)

o Unemployed looking for work (3)

o Unemployed not looking for work (4)

o Retired (5)

o Student (6)

o Disabled (7)

Q5 Education

o Less than high school (1)

o High school graduate (2)

o Some college (3)

o 2 year degree (4)

o 4 year degree (5)

o Professional degree (6)

o Doctorate (7)

Q6 Annual Household Income Level

o $0 - $24,999 (1)

o $25,000 - $49,999 (2)

o $50,000 to $74,999 (3)

o $75,000 to $99,999 (4)

o $100,000 to $149,999 (5)

o $150,000 to $199,999 (6)

o $200,000 or more (7)

Q7 US Citizenship

o US Citizen (1)

o No; pursuing US citizenship (2)

o No; not pursuing US citizenship (3)

Q8 International Citizenship

o Citizen of Nepal (1)

o Citizen of Bhutan (2)

o Other non-US citizenship (3)

o None (4)

Q9 Regardless of citizenship, I primarily identify as

o Nepalese (1)

o Bhutanese (2)

o American (3)

o Other (4)

Q10 What best describes you?

o Refugee (1)

o Immigrant (2)

o I was once a refugee, but no longer see myself as a refugee (3)

o None of these (4)

Q11 I have lived in the United States for ___ years.

________________________________________________________________

Q12 I have healthcare insurance.

o Yes, Private (1)

o Yes, Medicare/Medicaid (2)

o No (3)

o Unsure (4)

Q13 Religion

o Bhuddism (1)

o Christianity (2)

o Hindu (3)

o Islam (4)

o Kiratism (5)

o Other Religion (6)

o None (7)

Q14 I consider myself religious.

o Strongly disagree (1)

o Disagree (2)

o Neither agree nor disagree (3)

o Somewhat agree (4)

o Agree (5)

o Does not apply (6)

**End of Block: Demographics**

**Start of Block: Health/Healthcare Information**

Q15 I have ___ children.

________________________________________________________________

Q16 I have been pregnant ___ times in my life.

________________________________________________________________

Q17 I am currently pregnant

o Yes (1)

o No (2)

o Unsure (3)

Q18 My health is a priority in my life.

o Strongly disagree (1)

o Somewhat disagree (2)

o Neither agree nor disagree (3)

o Somewhat agree (4)

o Strongly agree (5)

Q19 I have access to healthcare.

o Strongly disagree (1)

o Somewhat disagree (2)

o Neither agree nor disagree (3)

o Somewhat agree (4)

o Strongly agree (5)

Q20 I personally have control over my health and healthcare decisions.

o Strongly disagree (1)

o Somewhat disagree (2)

o Neither agree nor disagree (3)

o Somewhat agree (4)

o Strongly agree (5)

| Page Break |  |
| --- | --- |

Q21 I have seen a doctor or advanced practice provider in the past year.

o Yes (1)

o No, but I've seen one in the past 3 years (2)

o No, but I've seen one in the past 5 years (3)

o No, but I've seen one in the past 10 years (4)

o No, but I've seen one more than 10 years ago (5)

o I've never seen a doctor or advanced practice provider (6)

Q22 I have specifically seen a gynecologist (OBGYN) or women's health specialist (NP, PA) in the past year.

o Yes (1)

o No, but I've seen one in the past 3 years (2)

o No, but I've seen one in the past 5 years (3)

o No, but I've seen one in the past 10 years (4)

o No, but I've seen one more than 10 years ago (5)

o I've never seen a gynecologist (6)

Q23 I only see a gynecologist (OBGYN) or women's health specialist (NP, PA) when I am pregnant or giving birth.

o Yes (1)

o No (2)

o Does not apply (3)

Q24 I only see a gynecologist (OBGYN) or women's health specialist (NP, PA) when there is something wrong.

▢	Yes (1)

▢	No (2)

▢	Does not apply (3)

Q25 I received a cervical/vaginal exam in the past year.

o Yes (1)

o No, but I've gotten one in the past 3 years (2)

o No, but I've gotten one in the past 5 years (3)

o No, but I've gotten one in the past 10 years (4)

o No, but I've gotten one more than 10 years ago (5)

o I've never received a cervical/vaginal exam (6)

o Unsure (7)

Q26 I received a breast exam or mammogram in the past year.

o Yes (1)

o No, but I've gotten one in the past 3 years (2)

o No, but I've gotten one in the past 5 years (3)

o No, but I've gotten one in the past 10 years (4)

o No, but I've gotten one more than 10 years ago (5)

o I've never received a breast exam or mammogram (6)

o Unsure (7)

Q27 I have been vaccinated against HPV (Human papillomavirus).

o Yes (1)

o No (2)

o Unsure (3)

Q28 I have had or currently have a diagnosis of cancer.

o Yes, Cervical Cancer (1)

o Yes, Breast Cancer (2)

o Yes, Breast and Cervical Cancer (3)

o Yes, Other Cancer (4)

o No (5)

o Unsure (6)

Q29 I've had a hysterectomy.

o Yes, total (1)

o Yes, partial (2)

o Yes, unsure which type (3)

o No (4)

Q30 I trust healthcare providers in the context of a well women's exam. (i.e., discussing sexual history, pregnancy history, receiving breast exams, vaginal/cervical exams).

o Strongly disagree (1)

o Somewhat disagree (2)

o Neither agree nor disagree (3)

o Somewhat agree (4)

o Strongly agree (5)

**End of Block: Health/Healthcare Information**

**Start of Block: Cultural/Spiritual Practices**

Q31 I participate or have participated in the practice Chhaupadi.

o Yes, but I no longer do because my beliefs have changed (1)

o Yes, but I no longer do because someone else's beliefs have changed (7)

o Yes, but I no longer do because it's not tradition in the US (2)

o Yes, but I no longer do because I no longer have periods (3)

o Yes, I currently practice Chhaupadi (4)

o No, never (5)

**End of Block: Cultural/Spiritual Practices**

**Start of Block: Understanding of Reproductive Cancers**

Q32 I understand what breast cancer is.

o Strongly disagree (1)

o Somewhat disagree (2)

o Neither agree nor disagree (3)

o Somewhat agree (4)

o Strongly agree (5)

Q33 I understand what cervical cancer is.

o Strongly disagree (1)

o Somewhat disagree (2)

o Neither agree nor disagree (3)

o Somewhat agree (4)

o Strongly agree (5)

Q34 I understand what HPV or human papillomavirus is.

o Strongly disagree (1)

o Somewhat disagree (2)

o Neither agree nor disagree (3)

o Somewhat agree (4)

o Strongly agree (5)

**End of Block: Understanding of Reproductive Cancers**

**Start of Block: Perception of Healthcare**

Q35 If I learned I had cancer, I believe it would be my fault.

o Strongly disagree (1)

o Somewhat disagree (2)

o Neither agree nor disagree (3)

o Somewhat agree (4)

o Strongly agree (5)

Q36 I could cure my own cancer through prayer or another spiritual method alone.

o Strongly disagree (1)

o Somewhat disagree (2)

o Neither agree nor disagree (3)

o Somewhat agree (4)

o Strongly agree (5)

Q37 I could cure my own cancer through lifestyle changes (diet, exercise, etc) alone.

o Strongly disagree (1)

o Somewhat disagree (2)

o Neither agree nor disagree (3)

o Somewhat agree (4)

o Strongly agree (5)

Q38 There is no need to treat cancer because it is deadly no matter what.

o Strongly disagree (1)

o Somewhat disagree (2)

o Neither agree nor disagree (3)

o Somewhat agree (4)

o Strongly agree (5)

| Page Break |  |
| --- | --- |

Q39 You should only get screened/checked for cancer if you have symptoms.

o Strongly disagree (1)

o Somewhat disagree (2)

o Neither agree nor disagree (3)

o Somewhat agree (4)

o Strongly agree (5)

Q40 It is useless to be checked for cancer because those cancers are deadly no matter what.

o Strongly disagree (1)

o Somewhat disagree (2)

o Neither agree nor disagree (3)

o Somewhat agree (4)

o Strongly agree (5)

Q41 I would be embarrassed if I was diagnosed with skin cancer.

o Strongly disagree (1)

o Somewhat disagree (2)

o Neither agree nor disagree (3)

o Somewhat agree (4)

o Strongly agree (5)

Q42 I would be embarrassed if I was diagnosed with breast cancer.

o Strongly disagree (1)

o Somewhat disagree (2)

o Neither agree nor disagree (3)

o Somewhat agree (4)

o Strongly agree (5)

Q43 I would be embarrassed if I was diagnosed with cervical cancer.

o Strongly disagree (1)

o Somewhat disagree (2)

o Neither agree nor disagree (3)

o Somewhat agree (4)

o Strongly agree (5)

Q44 I would be scared if I was diagnosed with skin cancer.

o Strongly disagree (1)

o Somewhat disagree (2)

o Neither agree nor disagree (3)

o Somewhat agree (4)

o Strongly agree (5)

Q45 I would be scared if I was diagnosed with breast cancer

o Strongly disagree (1)

o Somewhat disagree (2)

o Neither agree nor disagree (3)

o Somewhat agree (4)

o Strongly agree (5)

Q46 I would be scared if I was diagnosed with cervical cancer

o Strongly disagree (1)

o Somewhat disagree (2)

o Neither agree nor disagree (3)

o Somewhat agree (4)

o Strongly agree (5)

Q47 I feel adverse to screening for breast cancer (breast exam, mammogram).

o Strongly disagree (1)

o Somewhat disagree (2)

o Neither agree nor disagree (3)

o Somewhat agree (4)

o Strongly agree (5)

Q48 I feel adverse to screening for cervical cancer (Pap smear, vaginal exam).

o Strongly disagree (1)

o Somewhat disagree (2)

o Neither agree nor disagree (3)

o Somewhat agree (4)

o Strongly agree (5)

| Page Break |  |
| --- | --- |

Q49 I prefer female doctors/healthcare providers over male providers in general.

o Strongly disagree (1)

o Somewhat disagree (2)

o Neither agree nor disagree (3)

o Somewhat agree (4)

o Strongly agree (5)

Q50 I prefer female OBGYNs/women's health care providers over male providers.

o Strongly disagree (1)

o Somewhat disagree (2)

o Neither agree nor disagree (3)

o Somewhat agree (4)

o Strongly agree (5)

Q51 I would be more likely to consent to a breast or cervical exam from a female doctor.

o Strongly disagree (1)

o Somewhat disagree (2)

o Neither agree nor disagree (3)

o Somewhat agree (4)

o Strongly agree (5)

Q52 (Assuming I have my preferred doctor or provider) I want to stay modest, so I decline breast exams.

o Strongly disagree (1)

o Somewhat disagree (2)

o Neither agree nor disagree (3)

o Somewhat agree (4)

o Strongly agree (5)

Q53 (Assuming I have my preferred doctor or provider) I want to stay modest, so I decline cervical/vaginal exams.

o Strongly disagree (1)

o Somewhat disagree (2)

o Neither agree nor disagree (3)

o Somewhat agree (4)

o Strongly agree (5)

| Page Break |  |
| --- | --- |

Q54 Cervical exams (Pap smears) are painful. (Rate off of either experience or perception.)

o Strongly disagree (1)

o Somewhat disagree (2)

o Neither agree nor disagree (3)

o Somewhat agree (4)

o Strongly agree (5)

Q55 Vaginal exams (without a Pap smear) are painful. (Rate off of either experience or perception.)

o Strongly disagree (1)

o Somewhat disagree (2)

o Neither agree nor disagree (3)

o Somewhat agree (4)

o Strongly agree (5)

Q56 Breast exams are painful. (Rate off of either experience or perception.)

o Strongly disagree (1)

o Somewhat disagree (2)

o Neither agree nor disagree (3)

o Somewhat agree (4)

o Strongly agree (5)

Q57 Mammograms are painful. (Rate off of either experience or perception.)

o Strongly disagree (1)

o Somewhat disagree (2)

o Neither agree nor disagree (3)

o Somewhat agree (4)

o Strongly agree (5)

Q58 I have not scheduled a doctor’s appointment or have declined a breast exam because I want to avoid pain/physical discomfort from that exam.

o Strongly disagree (1)

o Somewhat disagree (2)

o Neither agree nor disagree (3)

o Somewhat agree (4)

o Strongly agree (5)

Q59 I have not scheduled a doctor’s appointment or have declined a mammogram because I want to avoid pain/physical discomfort from that exam.

o Strongly disagree (1)

o Somewhat disagree (2)

o Neither agree nor disagree (3)

o Somewhat agree (4)

o Strongly agree (5)

Q60 I have not scheduled a doctor’s appointment or have declined a vaginal exam (no Pap smear) because I want to avoid pain/physical discomfort from that exam.

o Strongly disagree (1)

o Somewhat disagree (2)

o Neither agree nor disagree (3)

o Somewhat agree (4)

o Strongly agree (5)

Q61 I have not scheduled a doctor’s appointment or have declined a cervical exam (Pap smear) because I want to avoid pain/physical discomfort from that exam.

o Strongly disagree (1)

o Somewhat disagree (2)

o Neither agree nor disagree (3)

o Somewhat agree (4)

o Strongly agree (5)

Q62 Finances prevent me from seeking medical care when something is wrong.

o Strongly disagree (1)

o Somewhat disagree (2)

o Neither agree nor disagree (3)

o Somewhat agree (4)

o Strongly agree (5)

Q63 Time constraints prevent me from seeking medical care when something is wrong.

o Strongly disagree (1)

o Somewhat disagree (2)

o Neither agree nor disagree (3)

o Somewhat agree (4)

o Strongly agree (5)

Q64 Finances prevent me from seeking medical care as a preventative measure.

o Strongly disagree (1)

o Somewhat disagree (2)

o Neither agree nor disagree (3)

o Somewhat agree (4)

o Strongly agree (5)

Q65 Time constraints prevent me from seeking medical care as a preventative measure.

o Strongly disagree (1)

o Somewhat disagree (2)

o Neither agree nor disagree (3)

o Somewhat agree (4)

o Strongly agree (5)

Q66 Language barriers make it difficult for me to receive healthcare.

o Strongly disagree (1)

o Somewhat disagree (2)

o Neither agree nor disagree (3)

o Somewhat agree (4)

o Strongly agree (5)

Q67 Before this survey, I was aware that women should receive regular cervical exams to prevent or find cancer.

o Definitely yes (1)

o Somewhat yes (2)

o Somewhat no (3)

o Definitely no (4)

Q68 Before this survey, I was aware that women should receive regular breast exams to prevent or find cancer.

o Definitely yes (1)

o Somewhat yes (2)

o Somewhat no (3)

o Definitely no (4)

**End of Block: Perception of Healthcare**

**Start of Block: Free Response**

Q69 Are there any cultural or spiritual beliefs you hold that you feel aren't considered at doctor's visits? Women's health visits specifically?

________________________________________________________________

Q70 Have you noticed a difference in women's healthcare internationally? What elements do you prefer from abroad? The US?

________________________________________________________________

Q71 Is there anything else you'd like for us to know about your experience with women's health exams? Remember, this survey is anonymous!

________________________________________________________________

**End of Block: Free Response**
